# Florivory indirectly decreases the plant reproductive output through changes in pollinator attraction

**DOI:** 10.1002/ece3.3921

**Published:** 2018-02-14

**Authors:** Kaoru Tsuji, Takayuki Ohgushi

**Affiliations:** ^1^ Center of Ecological Research Kyoto University Otsu Shiga Japan

**Keywords:** flower damage, flower size, fruit set, fruit size, seed set

## Abstract

Species often interact indirectly with each other via their traits. There is increasing appreciation of trait‐mediated indirect effects linking multiple interactions. Flowers interact with both pollinators and floral herbivores, and the flower‐pollinator interaction may be modified by indirect effects of floral herbivores (i.e., florivores) on flower traits such as flower size attracting pollinators. To explore whether flower size affects the flower‐pollinator interaction, we used *Eurya japonica* flowers. We examined whether artificial florivory decreased fruit and seed production, and also whether flower size affected florivory and the number of floral visitors. The petal removal treatment (i.e., artificial florivory) showed approximately 50% reduction in both fruit and seed set in natural pollination but not in artificial pollination. Furthermore, flower size increased the number of floral visitors, although it did not affect the frequency of florivory. Our results demonstrate that petal removal indirectly decreased 75% of female reproductive output via decreased flower visits by pollinators and that flower size mediated indirect interactions between florivory and floral visitors.

## 1 INTRODUCTION

Myriad species interact indirectly with each other in nature, which is known as “indirect effects.” Indirect effects are classified into two categories: density‐ and trait‐mediated. There is increasing evidence that trait‐mediated indirect effects can link multiple interactions (Ohgushi, 2005; Ohgushi, Schmitz, & Holt, 2012). In general, organismal traits are variable among individuals due to genetic, stochastic, and environmental effects (Doebeli, 1996; Fox & Kendall, 2002; Kendall & Fox, 2002). Also, some traits, such as plant‐induced defenses may be altered through interactions with other organisms (Poelman & Kessler, 2016). To what extent does the trait determine the strength of these trait‐mediated indirect interactions? This question has been explored by theoretical (Luttbeg, Rowe, & Mangel, 2003; Holt & Barfield, 2012), and empirical studies in predator‐prey systems (Ovadia & Schimitz, 2002; Matassa & Trusell, 2014; Gravem & Morgan, 2016) and in plant‐herbivore systems (Ohgushi, 2005; Freeman, 2006; Sendoya & Oliveira, 2015). These studies suggest that trait can change the strength of indirect interactions in a wide variety of systems.

To tackle this critical issue, flower‐insect interactions provide an excellent system. This is because flowers interact with not only pollinators but also floral herbivores (i.e., florivores), and flower traits such as flower size are critical to the strength of their interaction (McCall & Irwin, 2006). Furthermore, different pollinators and/or florivores respond to the floral traits in a species‐specific manner (Tsuji & Sota 2011, Tsuji & Sota, 2013; Antiqueira & Romero, 2016), and these floral visitors can affect each other (Romero, Antiqueira, & Koricheva, 2011; Fukano, Tanaka, Farkhary, & Kurachi, 2016), suggesting that the effect of floral traits on the floral visitors may be altered by species identity of floral visitors. Damage to floral tissues results in changes in flower size, shape, and nectar production (Krupnick & Weis, 1999; Strauss & Whittall, 2006). In this context, florivory can decrease fruit and seed set, either directly or indirectly via decreasing pollinator attraction (Krupnick & Weis, 1999; Mothershead & Marquis, 2000; Leavitt & Robertson, 2006; McCall & Irwin, 2006; Strauss & Whittall, 2006; Sánchez‐Lafuente, 2007; Carezza et al., 2011). Among a wide range of flower traits, flower size has been well studied showing that flower size can alter the strength of flower‐florivore and flower‐pollinator interactions (for florivores: McCall & Irwin, 2006; Teixido, Mendez, & Valladares, 2011; McCall & Barr, 2012; for pollinators: Willson, 1979; Bell, 1985; McCall & Irwin, 2006; Lobo, Ramos, & Braga, 2016; Sletvold & Agren, 2016). To examine whether flower size alters the strength of flower‐florivore‐pollinator interactions, we studied the interaction of *Eurya japonica* plants and its associated pollinators and florivores. As *E. japonica* has a long bud period of several months, florivory will likely occur before pollination. Florivores attacking *E. japonica* often consume stigmas and all petals (personal observation). Florivory on stigmas may directly decrease fruit and seed production and flower with a smaller size by florivory may indirectly decrease the reproductive output via decreasing pollinator attraction. Antiqueira and Romero (2016) reported that florivory lost floral symmetry of *Rubus rosifolius* and decreased pollinator attraction. However, it is unlikely to occur in *E. japonica*, because florivory to folded petals of flower buds does not cause asymmetry damage.

We first examined whether artificial damage before pollination directly and/or indirectly decreased fruit and seed production. Then, we examined whether bud size determined flower size because florivory occurred in bud period and floral visitors were attracted in blooming period. We examined whether the flower size affected florivory level and/or the number of visitors on blooming plant, based on the fact of a strong positive correlation between bud size and flower size.

## 2 MATERIALS AND METHODS

### 2.1 Study system

*Eurya japonica* (Pentaphylacaceae) is distributed across East Asia. This shrubby plant produces flower buds in summer, blooms in early spring, and bears mature fruits in autumn. Flowers are attacked by moth larvae including *Ourapteryx nivea, Alicis angulifera, Somena pulverea*, and *Chloroclystis excise* during bud and blooming stages (Tsuji & Sota, 2013), and these moth caterpillars damage approximately 3% of male flowers and <0.1% of female flowers (Tsuji & Sota, 2013). This male‐biased florivory seems to be due to defense chemicals in female sepals (Tsuji & Sota, 2010). Pollinators consist of generalist insects such as Diptera, Hymenoptera, and Coleoptera (Abe & Hasegawa, 2008), and fruits are dispersed by generalist birds (Chung & Kang, 1996; Manabe, Yamamoto, & Chiba,^1993^; Abe, Takahashi, & Hasegawa, 2014).

### 2.2 Field experiments

We conducted a field survey and experiments at Kozagawa in Wakayama prefecture, Japan. To examine how florivory affects fruit and seed production, we applied artificial florivory to female flowers for two reasons. First, female fitness can be easily and accurately estimated than male fitness (Conner, 2006). Second, the artificial florivory can make certain level of damage irrespective of defense of female flowers against florivores. We manipulated flowers of seven haphazardly selected female plants in 2012, and 26 female plants in 2013. We set four and seven treatments in 2012 and 2013, respectively.

#### 2.2.1 An experiment testing direct effect of floral damage on fruit set

**Figure 1:**
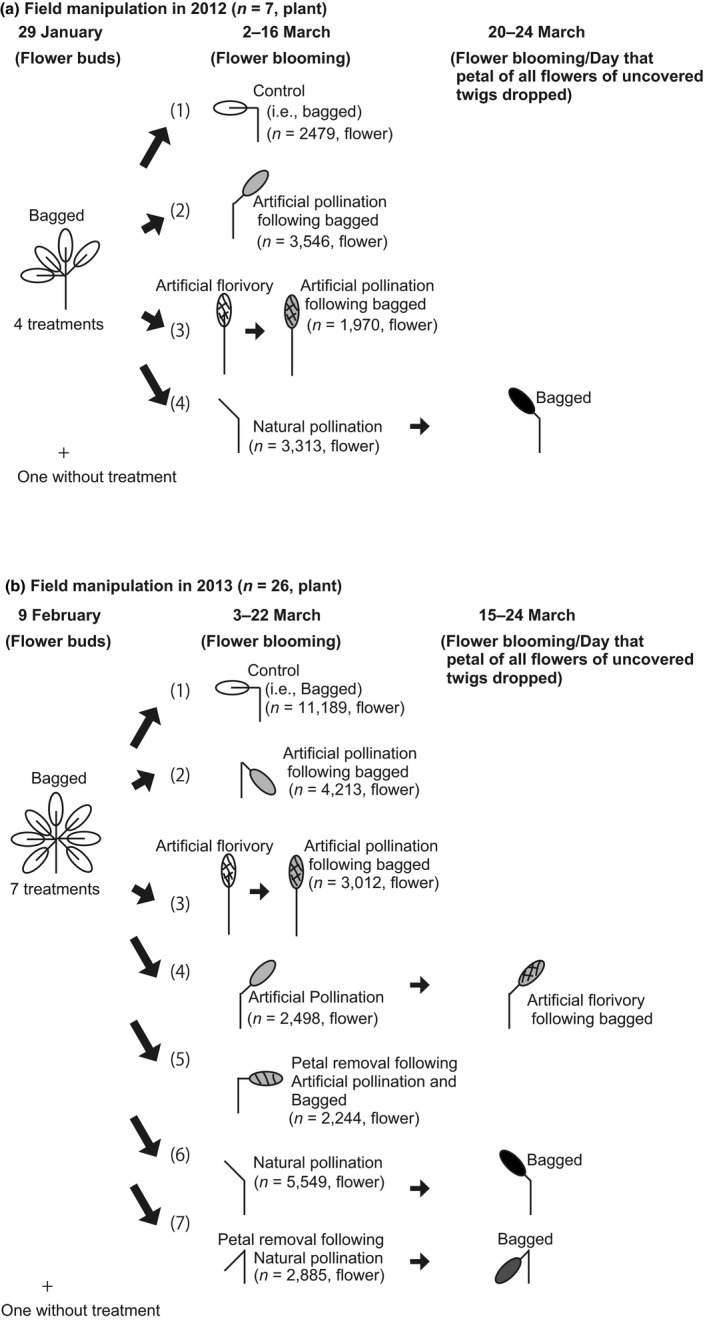
Experimental designs in 2012 and 2013

Seven plants have four treatments designed in a twig unit (Figure 1). On 29 January 2012, before blooming, we bagged all twigs with flower buds using nylon nets (mesh size: 0.46–0.59 mm). As damaged buds usually retain most of the ovules but not the stigmas, we removed stigmas to simulate florivory (i.e., artificial florivory [AF]). To examine how AF affects pollination, we set two treatments with artificial pollination (AP) from 2 to 16 March 2012 (i.e., one treatment had AP only and the other had AP after AF), using a brush and mixed pollen collected from five male plants around the female plants. AF and AP were conducted after flowers bloomed because of the difficulty of bud manipulation. We kept untreated and bagged twigs from pollination as a control (C). Then flowers on twigs were pollinated artificially (AP) and naturally (NP). Besides these three treatments, we set another treatment to evaluate the effect of artificial florivory before pollination (AF before AP). Four treatments were set as follows (Figure 1a):

1. No pollination (C): twigs were covered with nylon nets in the experimental period from 29 January to 19 May 2012.
2. Artificial pollination (AP)
3. Artificial florivory (AF) before AP
4. Natural pollination (NP): Net removal (NR) for pollination and bagged (B). Nets were removed for pollination on blooming days from 2 to 16 March. Then the twigs were bagged when all petals dropped from 20 to 24 March.

To evaluate fruit set, the number of fruits and dropped flowers in the nets was counted. On the same day, we measured the length of fruit on the treated twigs and another untreated twig (N) using a caliper.

#### 2.2.2 An experiment testing direct and indirect effect of floral damage on fruit and seed sets

In 2013, we bagged treated twigs on 9 February, just before flowering. To examine whether petal damage affects fruit set via changes in pollinator attraction, we removed all petals leaving stigmas intact (i.e., petal removal [PR]) using a pair of tweezers, as flowers lacking all petals were common following heavy florivory. Four treatments received artificial pollination (AP) from 3 to 24 March 2013. In addition to the same treatments in 2012, we conducted three additional treatments. To estimate the effect of the timing of florivory on female reproductive output, we removed stigmas after pollination. To examine the effect of petal damage on fruit and seed set, we removed petals of both artificially and naturally pollinated flowers. Seven treatments were as follows (Figure 1b):

1. No pollination (C): twigs were covered from 9 February to 6 August 2013.
2. Artificial pollination (AP)
3. Artificial florivory (AF) before AP
4. AP and AF following a few days later
5. Removal of all petals (PR) and AP on the same day
6. Natural pollination (NP): Net removal (NR) on the blooming day during the period from 3 to 12 March, exposure pollination for 6–16 days depending on respective twigs, and bagged (B) again on the day when petals of the twig dropped during the period from 15 to 24 March.
7. Removal of all petals (PR) exposed to natural pollination (NP): the nets of twigs were removed, followed by removal of all petals of flowers on the same day. Thereafter, the uncovered twigs were exposed to natural pollination for the same period for each twig as treatment six did, after which they were covered with the net again.

To examine fruit production, we counted the number of fruits and dropped flowers in the nets from 6 to 18 August 2013. During the same period, to examine seed production, we counted the number of developing seeds and dead ovules in fruits, and measured fruit length on the treated twigs and fruits on another untreated twig (N).

### 2.3 Measurement of bud and flower size

To confirm whether bud size determines flower size, we measured the size of buds and blooming flowers. We tagged 11 buds each of 15 male and 13 female plants using different color strings fastened on stalks on 4 January 2014 and then measured bud length using a caliper. Furthermore, we measured perianth length as flower size of the tagged flowers on the next day after flowers opened during the period from 13 February to 7 April 2015.

### 2.4 Record of flower damage

To examine whether bud size determines the proportion of buds with florivory, we checked bud damage and measured bud size of 13 male and 8 female plants on 2 January 2014. We counted the number of intact and damaged buds on ten 10 cm twigs of each plant. Finally, we measured the length of five buds of each plant using a caliper.

### 2.5 Sampling of floral visitors

To estimate the number of floral visitors around plants, we set 1–4 sticky traps (8 × 10 cm sheet; Gokiburi‐hoihoi Earth Chemical Co., Ltd.) on each of 17 male and 22 female plants from 12 to 14 March 2012. The traps were hung on twigs with twist ties 1–2 m above the ground. We directly counted the number of floral visitors that would have a function of pollination (Diptera, Hymenoptera, and Beetles) on the traps on 14 March.

Furthermore, we set 1–6 sticky traps on each of 18 male and 11 female plants on 19 and 23 March 2013. The traps were collected 24 hr later and soaked in canola oil overnight to detach insect samples. Collected samples were kept in 99% ethanol until 29 October 2013. The total number of floral visitors collected on 19 and 23 March was used for statistical analysis.

To confirm whether larger flowers receive more floral visitors, we recorded flower size of the plants for floral visitor sampling using a caliper. We measured the perianth length of 10–17 flowers and 6–11 flowers of each plant on 11 March 2012, 13 and 18 March 2013, respectively.

### 2.6 Statistical analysis

To compare fruit and seed set among treatments with considering individual variation, we applied generalized linear mixed models (GLMMs) with a negative binomial distribution and a log link function, as dispersion was too large to use Poisson distribution. We used the number of fruit/developing seeds as response variables. Model predictors included treatment and log‐transformed number of flowers/ovules. Plant individual was used as a random effect. GLMMs were performed using the package lme4 (Bates, Maechler, Bolker, & Walker, 2015), and were followed by likelihood ratio tests. Furthermore, to compare fruit and seed sets among treatments, we used pairwise tests (i.e., pairwise contrasts) using the packages lsmeans (Lenth, 2016) and multcomp (Hothorn, Bretz, & Westfall, 2008).

To test whether the fruit size was affected by treatments, we applied GLMMs with a gamma distribution and a log link function, followed by a chi‐square test and pairwise tests. In the 2012 experiment, fruit length, treatment, and plant individual were used as the response variable, explanatory variable, and random effect, respectively. In the 2013 experiment, we measured fruit size from 6 to 18 August 2013, and we included the measurement date as a fixed variable. To compare fruit size among treatments, GLMMs were followed by likelihood ratio test and pairwise tests. We also examined the association between developed seed number in a fruit and the size of fruit using a GLMM with a gamma distribution and a log link function followed by a chi‐square test. Fruit length, number of developed seed, and plant individual were the response variable, fixed explanatory variable, and random effect, respectively.

To examine the association between bud size and blooming flower size, we applied a GLMM with a gamma distribution and a log link function, followed by likelihood ratio tests. We used perianth length as the response variable. Bud length, plant sex and their interaction were explanatory variables, as sex significantly affected flower size (*df* = 2, Likelihood ratio = 22.8, *p *<* *.0001). Plant individual was included as a random effect.

To examine the effects of bud size on florivory, we used a generalized linear model (GLM) with a negative binomial distribution due to overdispersion and a log link function, using the MASS package (Venables & Ripley, 2002). The number of damaged buds was the response variable, and the number of observed buds, average bud length, plant sex, and their interactions were fixed explanatory variables. To test the significance of fixed variables, the GLM was followed by an *F‐*test using the car package (Fox & Weisberg, 2011).

To examine the effects of flower size on floral visitor number, we used GLMs with a negative binomial distribution and a log link function. We used the number of floral visitors as the response variable. Average of perianth length, number of traps on each plant, plant sex and interaction between perianth length and plant sex were fixed variables. As the number of traps was positively associated with the number of floral visitors, we also set the number of traps as a fixed variable. All statistical analyses were performed using R (version 3.3.1, The R Foundation for Statistical Computing Platform).

## 3 RESULTS

**Figure 2:**
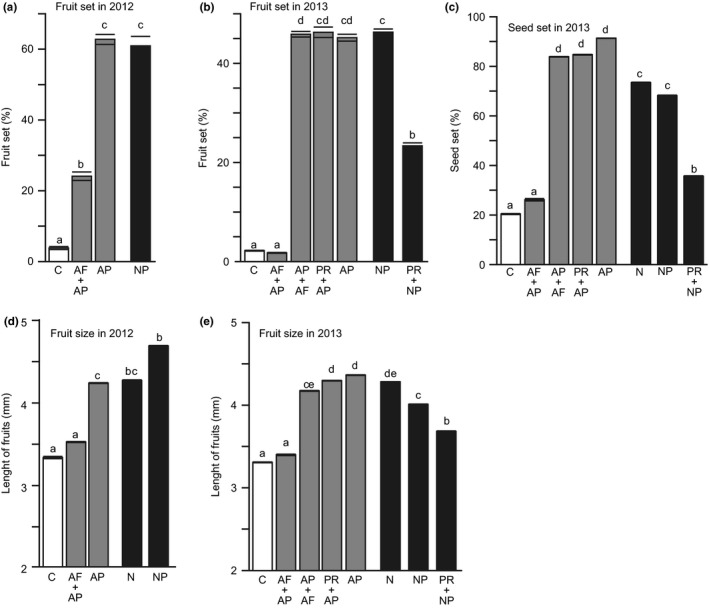
Fruit set (%) and size (mm) in 2012 and 2013 and seed set (%) in 2012. Abbreviations show treatments: control (C), artificial pollination (AP), artificial florivory (AF), petal removal (PR), natural pollination (NP), and unmanipulated (N). Different small letters on bars represent significant difference. Open, gray, and black bars represent unpollinated, artificially pollination, and natural pollination, respectively

Fruit size was positively correlated with the number of matured seeds within fruit (χ^2^_1_ = 1335.1, *p *<* *.0001). The experimental treatments significantly affected fruit set, fruit size, and seed set (Table 1a–c). Fruit set of flowers without pollination (C) and without stigmas (AF + AP) were significantly less than that of other treatments in both years (Figure 2a,b). However, the artificial florivory after artificial pollination (AP + AF), or petal removal after pollination (PR + AP), did not affect fruit set and seed set, compared to the artificially pollinated flowers (AP) in 2013 (Figure 2b,c). The naturally pollinated flowers without petals (PR + NP) produced significantly less fruits and seeds than those with petals (NP) in 2013 (Figure 2b,c). The effects of treatments on fruit size and seed set (Figure 2c–e) showed similar patterns to the effects on fruit set (Figure 2a,b).

**Table 1:** Effects of flower damage on fruit set and size and seed set

(a) Fruit set						
Factor	2012			2013		
	*df*	Likelihood ratio	*p*	*df*	Likelihood ratio	*p*
Number of pistils	1	18.9	**<.0001**	1	98.0	**<.0001**
Treatment	3	57.9	**<.0001**	6	273.5	**<.0001**

(b) Fruit size						
Factor	2012			2013		
	*df*	χ^2^	*p*	*df*	Likelihood ratio	*p*
Measured day	N.A.^a^			6	19.1	**.0040**
Treatment	4	319.3	**<.0001**	7	387.6	**<.0001**

(c) Seed set in 2013			
Factor	*df*	Likelihood ratio	*p*
Number of ovules	1	176.3	**<.0001**
Treatment	7	698.9	**<.0001**

a In 2012, day factor was not tested, as we measured on 1 day.

Bold letters indicate *p *<* *.05.

**Figure 3:**
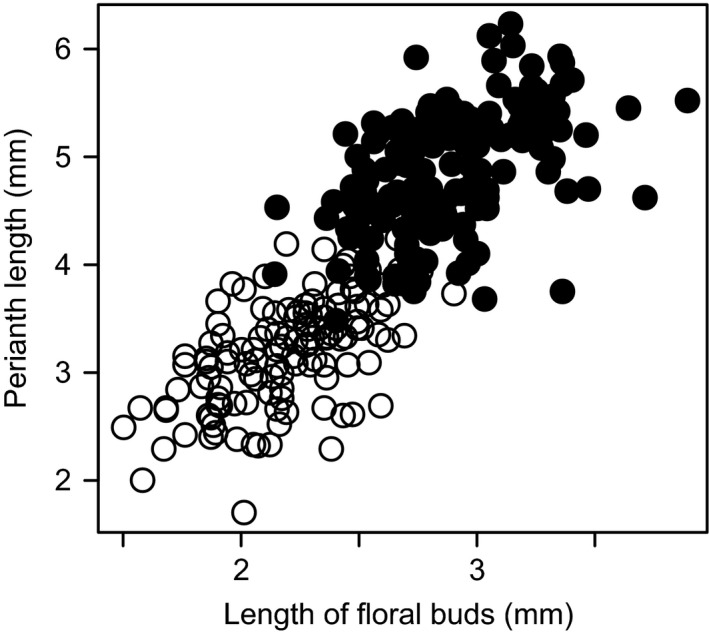
Relationships between floral bud length (i.e., bud size) and perianth length (i.e., flower size). Closed and open circles represent flowers of male and female plants, respectively

Bud size significantly increased with increasing blooming flower size (*df* = 2, Likelihood ratio = 50.4, *p *<* *.0001, Figure 3). Plant individual and sex significantly affected flower size (*df* = 1, 2, Likelihood ratio = 70.4, 22.8, *p *<* *.0001, <.0001, respectively), but their interaction did not (*df* = 1, Likelihood ratio = 0.7, *p *=* *.4). Although the proportion of buds with florivory was significantly affected by plant sex, it was not affected by bud size (Table 2).

**Table 2:** Effects of the size and number of buds and plant sex on the florivory

Factor	*F*_13,1_	*p*
Bud size	0.3	.60
Number of buds	0.5	.49
Plant sex	5.7	**.03**
Size × number	1.8	.21
Size × sex	0.0	1.00
Number × sex	0.0	1.00
Size × number × sex	0.0	1.00

Bold letters indicate *p *<* *.05.

The most abundant floral visitor was Diptera (99.1% and 75.5% in total visitors in 2012 and 2013, respectively), followed by Hymenoptera (0.7% and 21.6%), and Coleoptera (0.1% and 8.6%). The number of Diptera, including flies, was positively affected by flower size in 2012 (Table 3a). In 2013, the number of Hymenoptera increased with increasing flower size, although the number of Diptera was not significantly affected by flower size (Table 3b). Diptera in 2012 was significantly more abundant on male plants (*F*_34,1_ = 4.2, *p *=* *.048; 12 and 10 Diptera per trap on male and female plant, respectively). In contrast, Hymenoptera in 2013 was significantly more abundant on female plants (*F*_34,1_ = 7.4, *p *=* *.01). In 2012, the interaction between plant sex and flower size was significant for Diptera (Table 3a; *F*_34,1_ = 6.7, *p *=* *.01 for Diptera).

**Table 3:** Effect of flower size and plant sex on the number of floral visitors

(a) 2012									
Factor	Number of visitors		Flower size			Plant sex		Sex × size	
	Male plants (68 traps)	Female plants (73 traps)	Estimate of coefficient	*F*_34,1_	*p*	*F*_34,1_	*p*	*F*_34,1_	*p*
Total floral visitors	797	722	**1.22**	5.6	**.02**	4.3	**.046**	6.8	**.01**
Diptera	791	715	**1.39**	5.5	**.03**	4.2	**.048**	6.7	**.01**
Hymenoptera	5	6	−1.19	0.1	.73	0.1	.811	1.3	.26
Beetles	1	1	N.A.						

(b) 2013									
Factor	Number of visitors		Flower size			Plant sex		Sex × size	
	Male plants (118 traps)	Female plants (84 traps)	Estimate of coefficient	*F*_25,1_	*p*	*F*_25, 1_	*p*	*F*_25, 1_	*p*
Total floral visitors	201	182	2.86	0.3	.578	0.5	.48	0.4	.6
Diptera	155	134	0.97	0.2	.640	0.3	.58	0.0	1.0
Brachycera	96	74	1.50	0.2	.678	0.0	.97	0.0	.9
Muscoidea	66	50	2.10	0.3	.602	0.1	.76	0.0	1.0
Nematocera	59	60	−0.39	0.0	.841	0.9	.36	0.1	.8
Hymenoptera	22	39	**17.91**	4.4	**.047**	7.4	**.01**	1.3	.3
Beetles	24	9	−10.05	2.6	.120	4.6	**.04**	0.0	.9

Bold letters show significance (*p *<* *.05) for the estimates of partial regression coefficient.

## 4 DISCUSSION

Petal removal decreased both fruit and seed set by approximately 50% under natural pollination (Figure 2). This implies that petal removal results in 75% reduction ((1 − 0.5 × 0.5) × 100) in female reproductive output under natural pollination, although it did not decrease fruit and seed set when pollen was artificially supplemented (Figure 2). This suggests that petals are necessary to attract pollinators. As florivores badly destroy petals, damaged petals mediate the indirect interaction between florivores and pollinators. Furthermore, flower size increased the number of floral visitors (Table 3), although it did not affect the intensity of florivory (Table 2). As a result, flower damage by florivory decreases pollinator attraction and thus also female reproductive output.

### 4.1 Florivory indirectly decreases reproductive output

In the artificial florivory treatment, flowers that received stigma damage before pollination failed to produce fruits. However, flowers with stigma damage set fruit in 2012. This is because we may not have imposed sufficient damage to the stigmas. In 2013, as all stigmas were carefully removed, there was no fruit production by flowers without stigmas, which is consistent with the previous finding that damage of stigmas in *Chamerion angustifolium* inhibited pollination and thus decreased fruit set (Ladio & Aizen, 1999; Sheffield, Smith, & Kenan, 2005; Buchanan & Underwood, 2013). Thus, stigma damage of unpollinated flowers is a direct negative effect of florivory on fruit set. Interestingly, our results also suggest an indirect effect of florivory on fruit set; petal removal decreased fruit set when flowers were naturally pollinated, although it did not occur when flowers were artificially pollinated.

As well as fruit set, stigma damage directly and petal damage indirectly decreased seed set in 2013. Although we did not count seed in 2012, damaged flowers can be expected to have less seed. This is because damaged flowers resulted in smaller fruits than intact flowers in both 2012 and 2013, and the number of matured seed was positively related to fruit size.

The decrease in fruit and seed set suggests that petal damage will decrease pollinator attraction, thereby reducing female reproductive output, which is supported by studies in other plant‐pollinator systems (Karban & Strauss, 1993; Krupnick & Weis, 1999; McCall & Irwin, 2006; Strauss & Whittall, 2006; Carper, Adler, & Irwin, 2016).

### 4.2 Size effect of florivory and floral visitor attraction

Recent studies on how flower size affects florivory have suggested that larger flowers receive greater floral damage (McCall & Irwin, 2006; Teixido et al., 2011; McCall & Barr, 2012). In contrast, we did not detect the effects of bud size, which was positively correlated with flower size, on florivory. This implies that bud size does not affect the strength of flower‐florivore interactions.

On the other hand, this study showed that flower size was positively associated with the number of floral visitors. Also, the visitor number was significantly greater in male than female plants, and plant sex and the interaction between plant sex and flower size significantly affected the number of dipteran visitors (99.1% of total visitors) in 2012. These findings suggest that larger flowers are more preferred by insect pollinators, which is consistent with studies on other plant species (Willson, 1979; Bell, 1985; McCall & Irwin, 2006; Lobo et al., 2016; Sletvold & Agren, 2016).

## 5 CONCLUSION

Our study demonstrated that flower damage decreased female reproductive output via weakened flower visitor attraction, and this is consistent with studies showing that florivory decreases fruit and seed set indirectly via decreasing pollinator attraction (Krupnick & Weis, 1999; McCall & Irwin, 2006; Strauss & Whittall, 2006). In *E. japonica*, the petal removal decreased flower size and clearly decreased natural pollination. Furthermore, the low‐level of flower visitation can be caused by reduced petal size. Thus, the flower size is a critical trait that determines the strength of trait‐mediated indirect interactions among florivores, flowers, and pollinators. Also, note that different taxa responded to flower size differently, and their responses differed between years. As abundance and species composition of flower visitors can differ among years (Price, Waser, Irwin, Campbell, & Brody, 2005; Buide, 2006; Sgolastra et al., 2016), the indirect interaction mediated by flower size would temporally vary in its intensity, due to abundance and/or species composition of floral visitors.

## CONFLICT OF INTEREST

The authors have declared that no competing interests exist.

## AUTHOR CONTRIBUTIONS

KT conceived and designed this study, conducted the experiment, and analyzed the data. KT and TO contributed to the writing of the manuscript.
